# Changes in blood–spinal cord barrier permeability and neuroimmune interactions in the underlying mechanisms of chronic pain

**DOI:** 10.1097/PR9.0000000000000879

**Published:** 2021-03-09

**Authors:** Karli Montague-Cardoso, Marzia Malcangio

**Affiliations:** Wolfson Centre for Age-Related Diseases, Institute of Psychiatry, Psychology and Neuroscience, King’s College London, London, United Kingdom

**Keywords:** Blood–spinal cord barrier, Neuroimmune interactions, Monocytes/macrophages, Chemotherapy-induced pain

## Abstract

Advancing our understanding of the underlying mechanisms of chronic pain is instrumental to the identification of new potential therapeutic targets. Neuroimmune communication throughout the pain pathway is of crucial mechanistic importance and has been a major focus of preclinical chronic pain research over the last 2 decades. In the spinal cord, not only do dorsal horn neurons partake in mechanistically important bidirectional communication with resident immune cells such as microglia, but in some cases, they can also partake in bidirectional crosstalk with immune cells, such as monocytes/macrophages, which have infiltrated into the spinal cord from the circulation. The infiltration of immune cells into the spinal cord can be partly regulated by changes in permeability of the blood–spinal cord barrier (BSCB). Here, we discuss evidence for and against a mechanistic role for BSCB disruption and associated changes in neuroimmune crosstalk in preclinical chronic pain. We also consider recent evidence for its potential involvement in the vincristine model of chemotherapy-induced painful neuropathy. We conclude that current knowledge warrants further investigation to establish whether preventing BSCB disruption, or targeting the changes associated with this disruption, could be used for the development of novel approaches to treating chronic pain.

## Abstract 2

Here, we discuss evidence for changes in blood–spinal cord barrier permeability and consider the possibility of associated neuroimmune communication changes in models of chronic pain.

## 1. Introduction

Chronic pain is a distressing affliction that arises from different types of injury or disease. Patients with chronic pain conditions present with different manifestations, namely hyperalgesia (exaggerated response to noxious stimuli), allodynia (painful response to non-noxious stimuli), or spontaneous pain.^13^ Despite advances in our understanding of the underlying mechanisms of preclinical chronic pain, much remains unknown and current treatments can often possess limited efficacy or have a range of undesirable side effects.^2^ To develop novel, targeted treatments that could complement current therapies and thus improve treatment efficacy, without considerably contributing to the side-effect profile, we require a deeper understanding of the condition-specific mechanisms. Noxious stimuli are detected in the periphery by specialised sensory neurons, which have cell bodies located in the dorsal root ganglia and axons projecting centrally to their first synaptic contact with neurons in the dorsal horn of the spinal cord. Signals generated as a result of noxious stimuli are then transmitted to higher brain centres where pain is perceived. After peripheral nerve and tissue injury, the transition from acute to chronic pain involves changes in neuronal signalling throughout the pain pathway. Furthermore, there is a critical mechanistic importance for bidirectional communication between neurons and immune cells peripherally (at the site of injury and the dorsal root ganglia) and centrally.^4,20,34^ In the spinal cord for example, bidirectional communication between dorsal horn neurons and resident immune cells, such as microglia, has a well-established role in the underlying mechanisms of neuropathic and inflammatory pain.^37^ What has yet to be established, however, is the possible role of crosstalk between dorsal horn neurons and immune cells, which have infiltrated into the spinal cord from the circulation.

In this review, we discuss the preclinical evidence for the role of infiltrating immune cells into the spinal cord, which can sometimes occur when the blood–spinal cord barrier (BSCB) is disrupted. We find that in many preclinical chronic pain models, evidence for changes in BSCB permeability and immune cell infiltration are inconsistent and as such, a clear image remains elusive. We also discuss recent evidence for the direct actions of the chemotherapy agent vincristine, on endothelial cells that form the BSCB, which lead to mechanistically important changes in permeability and immune cell infiltration. We conclude by considering the therapeutic potential of targeting changes in BSCB permeability in the treatment of chronic pain and consider if further studies to develop such an approach are warranted.

### 1.1. Blood–spinal cord barrier structure and function: an overview

Spinal cord function is dependent on a precisely controlled homeostatic microenvironment, which is protected from the circulation by the BSCB. Although the BSCB is conceptually equivalent to the blood–brain barrier (BBB) and shares some of the same essential building blocks, there are crucial differences between the BSCB and BBB that result in them possessing distinct properties. For instance, the BSCB is more permeable than the BBB to cytokines, such as TNFα,^38^ as well as small tracers such as [^3^H]-D-Mannitol.^45^ The fundamental building blocks of the BSCB are nonfenestrated capillary endothelial cells, a basal lamina, and pericyte and astrocyte foot processes.^47^ The endothelial cells are connected by adhesion proteins and sealed by tight junctions, which play a key role in dictating barrier permeability.^1^ Some of the essential components of the tight junctions of the BSCB include the plasma membrane constituents claudin and occludin and the cytoplasmic protein constituent, zona occludens-1, or ZO-1.^48^ Their expression is reduced in the BSCB relative to the BBB, and this, in part, could account for the higher permeability of the former.^3^ In addition to reduced tight junction protein expression, in vitro studies indicate that the BSCB also possesses a reduced expression of adherens junction proteins, which are functionally linked with tight junctions.^17^ Furthermore, relative to the BBB, the BSCB also possesses lower expression of P-glycoprotein transporter, which serves as an efflux transporter and impedes drug penetration.^53^

Processes that result in the breakdown of BSCB components are associated with increased vascular permeability, changes in chemokines, and endothelial adhesion molecules to arrest the cells on the vessel wall and attract them into the spinal cord; these events will result in fundamental changes to the spinal cord microenvironment. There are several key proteins that regulate the breakdown of the BSCB. Matrix metalloproteinases (MMPs), for example, which degrade the extracellular matrix and extracellular proteins, also degrade components of the BSCB when their expression and activity is excessive.^36^ The role of MMPs varies. MMP-2, for example, plays a crucial role in wound healing,^26^ whereas MMP-3, 9, and 12 mediate BSCB breakdown.^29,36,55^ Indeed, in MMP-3 genetically deficient mice, both BSCB disruption and implications of this disruption including peripheral immune cell infiltration into the spinal cord, are significantly lower after spinal cord injury.^29^ In addition, the expression of occludin and ZO-1 are higher in MMP-3-deficient mice than controls.^29^ This indicates that MMP-3 is crucial for the breakdown of the BSCB after spinal cord injury. The infiltration of peripheral immune cells, such as monocytes/macrophages, is not only one of the potential consequences of BSCB disruption, but their presence can further steer the breakdown of the BSCB. Monocytes/macrophages are themselves a source of MMPs.^27,59^ MMP-12, for example, is released by macrophages and by promoting breakdown of BSCB components, becomes an important regulator of migration of immune cells across the endothelial basement membrane of the BSCB.^50,55^

In addition to MMPs, monocytes/macrophages can release the inflammatory cytokine TNFα, which, among many roles, can also regulate the breakdown of the BSCB. For example, within few hours after spinal cord compression, TNFα expression in the spinal cord is elevated, not only as a result of early BSCB opening, but also as a result of release from infiltrating monocytes/macrophages.^39,54^ One of the effects of elevated TNFα in the spinal cord was found to be an increase in BSCB permeability. This effect occurred through TNFα-mediated reduction in the expression of tight junction proteins ZO-1 and occludin, which is mediated through NF-kβ activation.^52^ Because of an effect on TNFα expression, bradykinin that is a vasodilator also plays an indirect role in regulating vascular permeability. However, although bradykinin antagonists, such as B9430, result in reduction in BSCB disruption, they do not affect injury-induced TNFα expression.^40^ Furthermore, macrophages also have the capacity to release endothelins, which are injury-dependent peptides that also play a role in injury-induced disruption of the BSCB.^28^ Immune cells such as monocytes/macrophages are therefore a source of proteins that can degrade components of the BSCB and thus disrupt permeability. It is important to note, however, that immune cell infiltration into the spinal cord and BSCB disruption are independent events, albeit overlapping, with immune cells also partaking in transcellular migration, which does not necessarily require BSCB disruption.^32^

In addition to proteins that regulate BSCB breakdown through an increase in expression or activity, the activity of other proteins is required to maintain the barrier and thus a decrease in their expression or activity results in breakdown of the BSCB. Angiopoietins, for example, are essential for the formation and maturation of blood vessels and for the survival of endothelial cells.^51^ In the event of spinal cord injury, angiopoietin 1 (Ang-1) expression is reduced^14^ and intravenous Ang-1 administration rescues blood vessels integrity and reduces permeability.^23^

There are thus many means by which BSCB permeability can be disrupted, and therefore, it is unsurprising that disruption of the BSCB occurs as part of, and plays a crucial role in, injury and diseases that predominantly affect the central nervous system, for example, traumatic spinal cord injury, amyotropic lateral sclerosis, and multiple sclerosis.^3^ Here, we consider the role that BSCB disruption could play in chronic pain models specifically.

### 1.2. Investigating blood–spinal cord barrier changes

Investigating blood barrier integrity in the central nervous system is a challenging process, which often involves a combination of techniques, thus it is important to become familiar with some of the methodologies that can be used.

One approach is to examine barrier morphology using techniques such as conventional and freeze-fracture electron microscopy and high-resolution immunohistochemistry. Such techniques give an indication of events such as mitochondrial degeneration in endothelial cells, changes in tight junction protein organization, swelling of astrocyte end feet, and trafficking of vesicles across endothelial cells—all of which are indicators that barrier integrity could be compromised.^5,16,41,44^ Although such an approach is useful and can indicate how the barrier may be compromised, it does not confirm how the function is altered, hence functional readouts, both in vitro and in vivo are also needed.

A well-established strategy for examining vascular permeability is the use of fluorescent tracers. In brief, tracers are injected intravenously before fluorescence imaging of spinal cord tissue.^56^ One of the earliest tracers used for this purpose is Evan's Blue (EB). Evan's Blue has a high affinity for serum albumin and forms a large complex of about 68 kDa which is unable to penetrate an uncompromised barrier. If the integrity of the BBB/BSCB is compromised, however, the EB-albumin complex can penetrate into the brain or spinal cord parenchyma, respectively. It can subsequently be visualised, either using light microscopy or fluorescence, with excitation peaks of 470 and 540 nm and emission at 690.^24,35^ Measuring EB is a reliable way to examine vascular permeability because of the high-affinity bond between EB and serum albumin, meaning that false positives are minimal. In addition to EB, protein luciferases are another group of fluorescent tracers that can be used in the same manner and provide similar information to EB in terms of the spatial and temporal profile of barrier disruption.^56^ When intravenously administered, luciferin undergoes oxidation, which results in the emission of enzymatic light that can be detected using excitation at 330 nm and emission at 530 nm. Alternatively, colorimetric luciferase assays can be used to measure expression in spinal cord tissue.^56^ In addition to fluorescent tracers, radiolabelled tracers, such as [^14^C]-alpha-aminoisobutyric acid are also commonly used to assess barrier permeability.^43^ Once injected intravenously, autoradiography can be used to detect and thence correlate levels of radioactivity in central nervous tissue and serum. As well as fluorescent and radioactive labels, paramagnetic contrast agents, such as gadopenetate dimeglumine, can also be injected intravenously and dynamic contract-enhanced magnetic resonance imaging is used to noninvasively and qualitatively measure barrier leakiness. Gadopenetate dimeglumine, for example, which has a relatively low molecular weight (938 Da), can penetrate the BSCB when it is compromised. This approach, because of its noninvasive nature, is particularly useful for examining temporal changes in permeability and has been used to assess changes after spinal cord injury.^12^ The enhancements that are visualised after injury are of 2 types—diffuse enhancements are caused by direct mechanical insult at the injury epicentre, whereas more focal enhancements are a result of decreases in BSCB permeability close to, but separate from the injury epicentre.

In general, any technique used to study vascular permeability in the CNS is useful but also limited. Specifically, those that provide insight into which of the BBB/BSCB building blocks are likely to be affected by injury do not tend to provide evidence for functional changes of the in vivo, whereas those which confirm functional changes in vivo do not give an indication of which components have been altered. The most effective approaches are therefore combinatorial.

### 1.3. Changes in blood–spinal cord barrier permeability and neuroimmune communication preclinical models of chronic pain

In peripheral nerve injury models of neuropathic pain, the occurrence and mechanistic importance of changes to BSCB permeability and infiltration of immune cells into the spinal cord, seems to be model-specific. In the case of inflammatory pain after tissue injury, findings are inconsistent between studies. For instance, both an increase in and lack of EB extravasation have been observed in the carrageenan inflammatory pain model. In this model, the onset of pain is within a matter of hours and usually peaks within the first 24 hours, to persists for at least 72 hours before resolving. Initial studies reported that within 48 hours of intraplantar carrageenan injection, an increase in EB extravasation in the spinal cord was observed, suggestive of BSCB disruption.^18^ However, more recent studies have been unable to observe the same changes at both 24 and 72 hours after the induction of inflammation.^15,58^ It is unlikely that issues surrounding the reliability of measuring EB account for the inconsistent results. Instead, a plausible explanation is that changes in BSCB permeability are transient and do not directly correlate with the development of pain in this model, which is consistent with changes in EB extravasation being highly dependent on the precise timepoint studied. Indeed, there are no apparent changes in BSCB permeability at 24 hours, when the pain-like behaviour is at its peak, but then a disruption of BSCB permeability occurs at 48 hours, only be to be resolved 24 hours later, at 72 hours. Although disruption of occludin morphology in the absence of EB extravasation has been reported at 72 hours,^58^ this is not confirmation of changes to tight junction function and thus BSCB permeability. Indeed, occludin protein expression was not altered at this time-point relative to controls.^58^ Although EB extravasation was absent at 72 hours, plasma IgG extravasation in the lumbar spinal cord of both male and female rats was observed^58^; this may indicate milder BSCB disruption at the late time-point or the engagement of an active process for IgG extravasation. Such a perturbation of BSCB integrity may underline the entrance of pronociceptive factors into the spinal cord that can facilitate nociceptive transmission.

In the case of preclinical neuropathic pain, there is more convincing evidence for BSCB disruption. Multiple studies have reported evidence for the occurrence of BSCB disruption in both the spared nerve injury (SNI) and chronic constriction injury (CCI) models. After both SNI in mice and CCI in rats, the BSCB has been found to be more permeable to different tracers including EB and sodium fluorescein (NaF) within the first 24 hours postinjury, when model-associated allodynia also manifests, but not at earlier timepoints.^6,49^ In addition to this, the expression of mRNA for tight junction proteins including occludin; claudin-1, claudin-5, and claudin-19; and ZO-1 was found to be significantly reduced between 7 and 14 days after CCI or SNI. In parallel, the number of pericytes (CD13^+^, platelet-derived growth factor receptor β^+^) were reduced. Pericytes are essential for the maintenance of the BSCB, and thus, an apparent reduction is indicative of a potential disruption to BSCB function.^49,57^ In the spinal nerve lesion model, evidence for longer-term changes to BSCB permeability has been obtained. Specifically, the greatest increase in leaked albumin and accompanying activation of astrocytes in the spinal cord was observed 2 weeks after injury but was still measurable as much as 8 weeks after this peak.^19^ Evidence for a precise mechanistic pathway by which the changes in BSCB permeability, however, has not yet been reported.

Dynamic contract-enhanced magnetic resonance imaging studies have provided some evidence that could indicate that the BSCB is disrupted in the SNI model of neuropathic pain, with a transient increase in permeability again being observed within the first 24 hours postinjury.^8^ This apparent BSCB disruption was relatively short-lived, however, with permeability of the BSCB returning to similar levels observed in control mice at time points later than 24 hours postsurgery. Interestingly in this study, although the extent of permeability varied between different mouse strains, it did not correlate with occurrence of neuropathic pain between strains. This indicates that BSCB disruption does not dictate the onset of nociception in this model and that crucial genetic determinants of injury-induced BSCB disruption are distinct from those that determine the genetic variability in peripheral nerve injury-associated hypersensitivity. Therefore, although the evidence for BSCB disruption in the model is convincing, evidence for immune cell infiltration and a mechanistic pathway underlying hypersensitivity is currently lacking. Indeed, in a recent study using a transgenic reporter line for peripheral immune cells (cx_3_cr1^GFP/+^ and Ccr2^RFP/+^), convincing evidence was obtained that indicated that they do not infiltrate the spinal cord parenchyma post-SNI.^22^

Another peripheral nerve injury model that has also been previously associated with changes in BSCB permeability is the sciatic nerve partial ligation (PNL) model. PNL has been found to result in long-term BSCB leakage, with both EB and NaF tracers being specifically present in the lumbar spinal cord as long as 4 weeks postsurgery.^15^ In this case, the suggested trigger of the leakage was found to be the pronociceptive cytokines MCP-1 and IL-1β, whereas in contrast, the antinociceptive cytokines IL-10 and TGF-β1 were found to “close” the openings created postinjury through changes in ZO-1 and occludin organisation. Critically, in this study, the leakage of the BSCB was accompanied by the recruitment of blood-borne monocytes/macrophages (identified using a GFP tag), which was prevented by intrathecal administration of TGF-β1. Although it is known that TGF-β1 exerts antinociceptive effects,^9^ the effect of intrathecal TGF-β1 administration on pain-like behaviour in this model was not examined, and, so, this alone is not indicative to suggest that there was a pronociceptive role. In addition, unequivocal evidence has since been obtained that demonstrates that although monocytes/macrophages play a crucial mechanistic role in regulating nociceptive signalling in neuropathic pain, specifically in surgical models, their infiltration into the spinal cord is absent. Indeed, changes in neuroimmune communication in the spinal cord in such models seem to require the responses of resident microglia and not blood-borne monocytes/macrophages. For example, after spinal nerve transection (SNT), pharmacological inhibition of microgliosis using cytosine arabinoside, reduced both microglial proliferation and SNT-associated hypersensitivity. However, both hypersensitivity and expansion of the spinal cord microglial population did not involve monocyte/macrophage infiltration.^21^

Blood-borne monocytes/macrophages can be differentiated from microglia by the expression of the chemokine receptor CCR_2_ and the purinergic receptor P2Y_12_. Monocytes/macrophages are positive for CCR_2_ yet negative for P2Y_12_, whereas conversely, microglia is negative for CCR_2_ and positive for P2Y_12_. After SNT, there was a lack of CCR_2_^+^/P2Y_12_^−^ monocytes/macrophages in the spinal cord demonstrating that the expansion of the microglial population in the spinal cord was not dependent on monocyte/macrophage infiltration. This does not indicate that peripheral monocytes/macrophages do not play a role in SNT-induced hypersensitivity. Indeed, they play a crucial role in the transition from acute to chronic pain.^42^ However, the infiltration of monocyte/macrophages into the spinal cord specifically seems to be absent. This however does not indicate that BSCB disruption had not occurred in this model as BSCB disruption is not always accompanied by immune cell infiltration. However, this data set is nonetheless a strong indicator that infiltration of peripheral monocytes/macrophages into the spinal cord, and thus, changes in monocyte/macrophage-mediated neuroimmune crosstalk are not a downstream consequence of potential BSCB changes. Instead, BSCB disruption could result in the extravasation of smaller molecules such as cytokines that exert pronociceptive effects or may result in the infiltration of other immune cells such as T cells.

Indeed, recent evidence has been obtained that indicates that BSCB disruption and T-cell infiltration could play an important role in regulating pain-like behaviour in a model-specific manner. For instance, when CCI was performed in rats, measurements of NaFlu concentration in the spinal cord confirmed that an increase in BSCB permeability was likely to occur in this model and in this case, in the lumbar region specifically.^31^ Furthermore, in parallel to BSCB disruption, an increase in CD3^+^ T cells in the spinal cord parenchyma was observed, along with CXCL_10_/CXCR_3_ signalling pathway activation, which is known to promote the infiltration of peripheral T cells into the spinal cord.^31,48^ Critically, intrathecal administration of a neutralising antibody against CXCL_10_ not only prevented disruption of the BSCB but also reduced hyperalgesia, indicating that, at least in part, both disruption of the BSCB and CXCR_3_-expressing T cells-mediated effects, play a crucial mechanistic role in neuropathic pain in this model.

Taken together, there is convincing evidence for BSCB disruption in nerve injury models of neuropathic pain. Infiltration of blood-borne immune cells, however, is not always a consequence, and further studies are needed to precisely determine the broad downstream effects of BSCB disruption.

### 1.4. Blood–spinal cord barrier disruption and changes in neuroimmune communication in chemotherapy-induced pain

Although changes in BSCB permeability in surgical models of neuropathic pain have been studied over the last decade, evidence has only recently been obtained to suggest the presence of, and a crucial mechanistic role for, BSCB disruption and accompanying monocyte/macrophage infiltration into the spinal cord in the vincristine (VCR) model of chemotherapy-induced neuropathic pain. Specifically, in vivo, within as little as 24 hours after VCR systemic administration, intravital microscopy revealed that inflammatory monocytes (CCR_2_^+^) had begun to infiltrate into the spinal cord, which could be indicative of changes in BSCB permeability.^35^ Indeed, after one cycle of VCR treatment (5 days) in mice, not only was EB extravasation into the spinal cord significantly elevated relative to controls, but immunohistochemical evidence revealed both disorganisation of claudin-5 and ZO-1 and an accompanying reduction in their expression.^35^ In addition, in the lumbar spinal cord of mice treated with a cycle of VCR, there was an increase in the protein expression of the protease cathepsin S (CatS) and a presence of monocytes/macrophages (Ly6C^+^ cells, which were also negative for the marker for resident microglial, TMEM119), which were positive for CatS. In other words, infiltrated monocytes/macrophages could have plausibly accounted for the increase in CatS expression in the spinal cord. CatS has well-established pronociceptive effects by its cleavage of neuronal fractalkine,^11^ which results in the generation of soluble fractalkine, which in turn activates CX_3_CR_1_ receptors on microglia and thence regulates the release of pronociceptive cytokines.^36^ Thus VCR-induced BSCB disruption could have facilitated the infiltration of CatS^+^-inflammatory monocytes/macrophages, which exert pronociceptive effects through release of this protease. Indeed, critically, administration of a centrally penetrant CatS inhibitor in VCR-treated animals significantly reduced allodynia, whereas a peripherally restricted inhibitor had no effect on withdrawal thresholds.^35^ This highlights the importance of spinal cord expression of CatS over that found peripherally in this model of chemotherapy pain and therefore the potential mechanistic importance of BSCB disruption and monocyte/macrophage infiltration into the spinal cord, which could be a direct result of such disruption. A plausible explanation for the effect of a chemotherapy agent such as VCR on the BSCB is the direct activation of the endothelium. Specifically, in vitro, VCR treatment activated endothelial cells within 24 hours of application, consequentially increasing paracellular permeability of reducing transendothelial resistance.^35^ Interestingly, other chemotherapy agents have also been found to have the capacity to target endothelial cells. Itraconazole, which has recently been repurposed for cancer treatment, for example, inhibits the proliferation of endothelial cells.^10^ In addition, oxaliplatin, which also induces allodynia^25^ disrupts ZO-1 expression in CNS endothelial cells in vitro as well as inducing the expression of reactive oxygen species and endoplasmic reticulum stress—all of which would disrupt endothelial cells function and then vascular permeability.^7^

**Figure 1.**
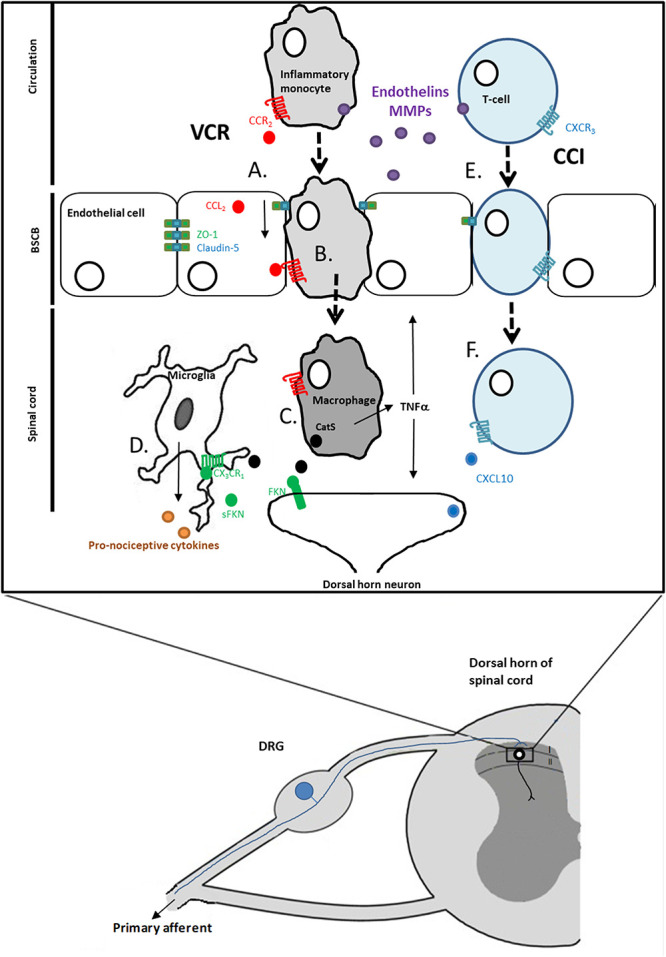
Blood–spinal cord barrier disruption and immune cell infiltration into the spinal cord as an underlying mechanism of neuropathic pain. (A) One downstream effect of treatment with chemotherapy agents such as vincristine (VCR) is the activation of endothelial cells of the BSCB and disruption of tight junctions. (B) CCR_2_^+^ monocytes infiltrate into the spinal cord in response to the release of CCL_2_ from endothelial cells. (B) Monocytes/macrophages release CatS, which cleaves neuronally expressed fractalkine (FKN) producing soluble fractalkine (sFKN). (D) sFKN activates CX_3_CR_1_ receptors on microglia, which in turn release pronociceptive mediators. (E) After chronic constriction injury (CCI) there is evidence for BSCB disruption. (F) One outcome of such disruption is that CXCR_3_-expressing T-cells infiltrate into the spinal cord.

Taken together, the downstream effects of changes in BSCB permeability are very much model-dependent. For instance, although it currently seems unlikely that BSCB disruption leads to infiltration of monocytes/macrophages in models of inflammatory pain and surgical models of neuropathic pain, there is evidence to suggest that it is an underlying mechanism in the VCR chemotherapy pain model. In the CCI model, however, it currently appears that mechanisms mediated by infiltrating T cells may play an important role in neuroimmune communication in the spinal cord after BSCB disruption (Fig. 1). Targeting the signalling of monocytes/macrophages and T cells that have infiltrated into the spinal cord after chemotherapy and surgical injury, respectfully, could provide a new therapeutic avenue to consider. In both cases, however, advances in our tools and understanding of the cellular processes are needed, and it is crucial to remember that BSCB disruption and immune cell infiltration are independent events. Nonetheless, in cases where BSCB disruption is accompanied by immune cell infiltration for instance, it is important to be mindful of the fact that although the latter may very well be a direct consequence of the former, this is not guaranteed to be the case.

### 1.5. Clinical considerations

Although it seems that disruption of the BSCB is a feature of preclinical neuropathic pain, to contemplate the potential translational value, in the future it will be vital to establish if such a phenomenon is present in patients with chronic pain. Interestingly, patients with CNS diseases in which the BSCB is known to be disrupted, such as amyotropic lateral sclerosis, also report chronic pain as a comorbidity.^46^ This could suggest that changes to BSCB permeability manifest in pain; however, the multifactorial nature of CNS diseases means that a direct, causative relationship between changes in BSCB permeability and chronic pain in the clinic is difficult to establish and as a result studies are predominantly correlative at present.

In terms of identifying changes in BSCB permeability in chronic pain patients, the most useful approach is likely to be the use of magnetic resonance imaging studies, which have been used to assess spinal cord structural abnormalities in multiple sclerosis patients.^33^ Should BSCB changes be confirmed, the potential therapies considered could tackle the breakdown of the BSCB directly, for example, by targeting MMPs. Therapies, which tackle the breakdown of the BSCB and are already clinically available, include the use of fluoxetine in conjunction with vitamin C supplements, which has been found to inhibit MMPs.^30^ However, the effects are nonspecific and thus unwanted side effects are unavoidable. Therefore, targeting precise signalling pathways, such as the inhibition of CatS in VCR-induced pain,^35^ could constitute the basis of a more appealing approach. Data obtained to date regarding the role of BSCB disruption and immune cell infiltration in chronic pain are very much in its infancy and by no means conclusive. Therefore, further studies along this line of investigation are warranted.

## 2. Summary

In this review, we discuss the preclinical evidence for the role of changes in permeability of the BSCB in models of chronic pain. We explain that, at present, although there is convincing evidence for disruption of the BSCB in neuropathic pain, the downstream consequences seem to be model-specific. For example, there is some evidence that T cells may infiltrate the spinal cord after CCI-induced BSCB disruption and release pronociceptive factors, whereas in the case of VCR treatment, BSCB disruption could result in monocyte infiltration and thence release pronociceptive signals such as CatS. Clinically, in some diseases in which the BSCB is disrupted, pain is reported as a common comorbidity, however, given the multifactorial nature of such diseases, a direct link between BSCB disruption and pain is still lacking. The notion of targeting BSCB disruption or its consequences to treat chronic pain is very much in its infancy and as a result, clinical promise has yet to be determined.

## Disclosures

The authors have no conflicts of interest to declare.
